# ADAMTS13 ameliorates inflammatory responses in experimental autoimmune encephalomyelitis

**DOI:** 10.1186/s12974-020-1713-z

**Published:** 2020-02-19

**Authors:** Kaili Lu, Lan Liu, Xiaofeng Xu, Fei Zhao, Jiangshan Deng, Xin Tang, Xiuzhe Wang, Bing-Qiao Zhao, Xiaojie Zhang, Yuwu Zhao

**Affiliations:** 1 Department of Neurology, Shanghai Jiao Tong University Affiliated Sixth People’s Hospital, No. 600, Yishan Road, Xuhui District, Shanghai, China; 2 Department of Translational Neuroscience, Jing’an District Centre Hospital of Shanghai, State Key Laboratory of Medical Neurobiology and MOE Frontiers Center for Brain Science, Institutes of Brain Science, Fudan University, Shanghai, China

**Keywords:** Multiple sclerosis, Experimental autoimmune encephalomyelitis, ADAMTS13, VWF, Inflammation

## Abstract

**Background:**

ADAMTS13 (a disintegrin and metalloprotease with a thrombospondin type 1 motif, member 13) plays a vital role in preventing microvascular thrombosis and inflammation. Reduced ADAMTS13 levels in plasma have been detected in multiple sclerosis (MS) patients. In the present study, we have determined the role of ADAMTS13 in the disease progression of MS using a mouse model of experimental autoimmune encephalomyelitis (EAE).

**Methods:**

Female C57BL/6 mice were immunized with MOG_35–55_ peptide and then treated with ADAMTS13 or vehicle in preventive and therapeutic settings. Mice were analyzed for clinical deficit, white matter demyelination and inflammatory cell infiltration. To explore the underlying mechanism, VWF expression and blood-spinal cord barriers (BSCB) were determined.

**Results:**

Plasma ADAMTS13 activity was suppressed in EAE mice. ADAMTS13-treated EAE mice exhibited an ameliorated disease course, reduced demyelination, and decreased T lymphocyte, neutrophil and monocyte infiltration into the spinal cord. Consistently, ADAMTS13 treatment reduced VWF levels and inhibited BSCB breakdown in the spinal cords of EAE mice. However, leukocytes in the blood and spleen of EAE mice remained unaffected by ADAMTS13 administration.

**Conclusion:**

Our results demonstrate that ADAMTS13 treatment ameliorates inflammatory responses, demyelination and disease course in EAE mice. Therefore, our study suggests that ADAMTS13 may represent a potential therapeutic strategy for MS patients.

## Introduction

Multiple sclerosis (MS) is an inflammatory demyelinating disease of the central nervous system (CNS), that is characterized by leukocyte infiltration and myelin damage [1, 2]. As an animal model for MS, experimental autoimmune encephalomyelitis (EAE) has been widely used to study the pathophysiology and therapy of MS. It is commonly acknowledged that MS and EAE are mediated by autoreactive T lymphocyte cells that enter the CNS and initiate a chronic autoimmune response [3].

Recent years have seen an increasing relationship between hemostasis and inflammation. Cells and proteins of the hemostatic system, traditionally studied in thrombotic diseases, clearly play unexpected roles in MS and EAE. For example, tissue factor and protein C inhibitor were detected within the chronic active lesions MS patients by proteomic analysis [4]. In addition, thrombin and fibrinogen [5, 6] proteins were shown to promote CNS inflammation in MS. In addition, coagulation factor XII deficiency [7] and anticoagulation treatment with warfarin or rivaroxaban [8] were reported to ameliorate EAE injury by modulating inflammatory responses.

Interestingly, a recent study assessed plasma samples from MS patients and matched healthy individuals (HI) by magnetic Luminex assays and enzyme-linked immunosorbent assay (ELISA). Among the plasma hemostasis factors, ADAMTS13 levels were significantly decreased in MS patients [9]. ADAMTS13, a metalloprotease that is able to cleave von Willebrand factor (VWF), was first discovered in thrombotic thrombocytopenic purpura (TTP) [10, 11]. In addition, preclinical studies of ADAMTS13 have shown promise in reducing the inflammatory response of various neurological disease models such as ischemic and hemorrhagic stroke [12–16]. Thus we were inspired to assess the potential influence of ADAMTS13 therapy on autoimmune inflammation in chronic EAE mice.

## Methods

### Animals

C57BL/6 female mice (18–20 g) were obtained from Shanghai Sipper-BK Laboratory Animal Corp. Ltd. Mice were bred and kept with a 12 h light–dark cycle, with free access to food and water under specific pathogen-free conditions. The study was approved by the Shanghai Jiao Tong University Affiliated Sixth People’s Hospital Animal Ethics committee.

### EAE induction and scoring

Briefly, 200 μg myelin oligodendrocyte glycoprotein 35–55 (MOG_35–55_) peptide (GL Biochem Corporation. Ltd., Shanghai) dissolved in 100 μl phosphate-buffered saline (PBS) was emulsified with 100 μl of complete Freund’s adjuvant (Sigma, St Louis, MO) supplemented with 400 μg of *Mycobacterium tuberculosis* H37Ra (Difco Laboratories, Detroit, MI). Then, 8-week-old mice were anesthetized by isoflurane and then immunized with the above emulsion via subcutaneous injection on day 0. Pertussis toxin (Merck KGaA, Darmstadt) was administered intraperitoneally at 0 and 2 days post-immunization (dpi).

Clinical scores were monitored daily in a blind manner. Mice were scored on a scale of 0–5 based on the degree of ascending paralysis [17]: 0, no symptoms; 0.5, partial limp tail; 1, complete limp tail; 1.5, hind limb ataxia; 2, hind limb paresis; 2.5, partial hind limb paralysis; 3, complete hind limb paralysis; 3.5, hind limb paralysis and fore limb paresis; 4, hind and fore limb paralysis; 5, moribund.

### Analysis of plasma ADAMTS13 activity and VWF multimer

Blood was obtained from mice at different times post-EAE immunization and stored in tubes with 3.8% sodium citrate (at a ratio of 9:1 vol/vol). After centrifugation at 3000 g for 20 min, plasma was stored at − 80 °C until analysis.

ADAMTS13 activity in plasma was determined using FRETS-VWF73 peptide (Peptides International) as previously described [18]. Briefly, FRETS-VWF73 was incubated with plasma in reaction buffer (5 mM Bis-Tris, 25 mM CaCl_2_, 0.005% Tween-20 [pH 6.0]). Fluorescence intensities were detected every 5 min for 1 h with a fluorescence spectrophotometer (Bio-Tech) using excitation at 340 nm and emission at 450 nm. The analysis of ADAMTS13 activity in the cerebrospinal fluid (CSF) and spinal cords of mice with commercially available FRETS-VWF73 peptide was not satisfactory, and thus these analyses were precluded from our study.

The plasma VWF multimer was analyzed as previously described [18–20]. Mouse plasma (4 μl) was diluted in 70 mM Tris-HCl buffer (16 μl), pH 6.5 containing 2.4% sodium dodecyl sulfate, 4% urea, and 4 mM EDTA and then heated at 60 °C for 20 min. The sample (20 μl) was fractionated on a 1.2% SeaKem HGT agarose mini-gel (Lonza) by electrophoresis and transferred onto a nitrocellulose membrane (Merck KGaA). The membrane was incubated with rabbit anti-human VWF antibody (DAkO) and then detected with horseradish peroxidase-conjugated anti-rabbit IgG. The signal was obtained using an ImageQuant LAS 4000 mini system (GE Healthcare).

### ADAMTS13 treatment

Previous studies have confirmed the enzymatic activity of recombinant human ADAMTS13 in mice [21]; thus, recombinant human ADAMTS13 (R&D systems) was adopted. Four days before ADAMTS13 treatment, mice were anesthetized by isoflurane, and a 26-gauge stainless steel guide cannula was implanted into the lateral ventricles (0.2 mm posterior to bregma and 0.9 mm lateral to midline). In the preventive setting, a total of 50 EAE mice were randomly divided into two groups: vehicle group and ADAMTS13 group. Vehicle (2 μl sterile phosphate-buffered saline, PBS) or ADAMTS13 (50 ng in 2 μl PBS) was delivered into the lateral ventricles daily from 7 dpi to 21 dpi. During the injection period between 7 dpi and 21 dpi, the mortality rate was 8%. To test the therapeutic effect, ADAMTS13 (50 ng in 2 μl PBS) or vehicle (2 μl sterile PBS) was injected into the lateral ventricle of EAE mice for 15 days since the clinical score reached 1 (15 dpi).

### Tissue preparation

On 22 and 30 dpi, mice were euthanized by pentobarbital. Then, mice were transcardially perfused with 0.1 M PBS and fixed with 4% paraformaldehyde (PFA). Lumbar spinal cords were resected, postfixed overnight in 4% PFA, and then cryoprotected in 20% and 30% sucrose solution at 4 °C. Spinal cord sections were embedded in Tissue-Tek optimum cutting temperature (OCT) compound (Sakura Finetek, Torrance, CA, USA) and cut into 10-um-thick transverse sections with a freezing microtome (Leica CM1950).

### Histopathology

Spinal cord sections were stained with luxol fast blue (LFB) for the detection of demyelination and hematoxylin and eosin (H&E) for the assessment of inflammation. Briefly, sections were incubated with 0.1% LFB (Sigma, St Louis, MO) at 60 °C overnight and differentiated in 0.1% lithium carbonate solution and 70% ethyl alcohol. Quantification of demyelination was performed by tracing the areas resistant to the LFB stain and dividing the area by the total white matter area using Image-Pro Plus software. H&E stained sections were scored in a semiquantitative fashion for inflammation: 0, none; 1, a few inflammatory cells; 2, organization of perivascular infiltrates; and 3, perivascular cuffing with extension into the adjacent tissue [22]. For quantification of LFB and HE, four to five serial sections from every mouse were examined.

### Immunofluorescence

For immunofluorescence, spinal cord sections from EAE mice were incubated in blocking solution (3% donkey serum in PBS) containing 0.3% Triton X-100 at room temperature for 30 min. Then, sections were stained overnight at 4 °C with primary anti-CD3e (R&D systems), anti-Iba-1 (Abcam), anti-Ly6G (BD Biosciences), and anti-VWF (DAKO) antibodies. Alexa Fluor 488-coupled donkey antibodies (Abcam) or Alexa Fluor 555-coupled donkey antibodies (Abcam) were used as secondary antibodies, and DNA in the nucleus was stained with DAPI (CST). Sections were imaged by fluorescence microscopy (IX53, Olympus). The analyses of immune cell markers (CD3 and Ly6G) were quantified by the number of positive cells per field, a 200 μm × 150 μm rectangle. For analysis of Iba-1 mean fluorescence intensity with Image-Pro Plus software, all sections were immunolabeled and collected as a single batch using the same parameters. For every EAE mouse, the values were averaged from 15 fields covering the white matter of three spinal cord sections.

### Flow cytometry

In a preventive setting at 22 dpi, blood was collected in EDTA-coated tubes via cardiac puncture in mice overdosed with sodium pentobarbital. Spleens were ground and passed through a 70-μm nylon mesh cell strainer (BD Biosciences) to obtain a single cell suspension for further processing. Spinal cords were harvested from PBS-perfused mice and then ground and filtered through a 70-μm strainer. Leukocyte cells were isolated by 30% and 70% Percoll gradient (GE Healthcare).

FITC-conjugated anti-CD3e, PerCP-Cy5.5-conjugated anti-CD4, PerCP-Cy5.5-conjugated anti-CD11b, APC-conjugated anti-CD45, PE-conjugated anti-Ly6G, PE-Cy7-conjugated anti-Ly6C, PE-conjugated anti-IL-17A, and APC-conjugated IFN-γ antibodies were purchased from eBioscience. To analyze inflammatory cells in EAE mice, cells isolated from the blood, spleen, and spinal cord were blocked with anti-CD16/CD32 mix (BD Biosciences) and then stained with anti-CD45, anti-CD11b, anti-CD3e, anti-Ly6G, and anti-Ly6C antibodies on ice for 30 min. Zombie Violet™ (Biolegend) was used to distinguish live from dead cells. For analysis, doublets and dead cells were discarded, and leukocytes were identified as follows: T lymphocytes (CD45^hi^ CD11b^−^ CD3^+^), microglia (CD45^mild^ CD11b^+^), neutrophils (CD45^hi^ CD11b^+^ Ly6C^int^ Ly6G^hi^), and monocytes (CD45^hi^ CD11b^+^ Ly6C^hi^ Ly6G^−^). Among these CD45^hi^ leukocytes infiltrated into spinal cords, T lymphocytes, neutrophils, and monocytes were pre-gated on CD45^hi^ cells.

For intracellular staining, cells from spleens were harvested and stimulated in complete media (RPMI media containing 10% FCS) for 5 h at 37 °C with Cell Stimulation Cocktail (plus protein transport inhibitors) (eBioscience). Subsequently, cells were stained with anti-CD3e and anti-CD4 antibodies, fixed and permeabilized using Fixation and Permeabilization Buffer Set (eBioscience), and then stained with anti-IL-17A and anti-IFN-γ antibodies. Stained cells were acquired using a CytoFLEX flow cytometer (Beckman), and data were analyzed with CytExpert software (Beckman).

### Analysis of blood-spinal cord barriers

At 22 dpi, mice were randomly selected for assessment of blood-spinal cord barrier (BSCB) permeability using the Evans blue extravasation method. Briefly, 2% Evans blue (EB; Sigma, St Louis, MO) dissolved in saline was intravenously injected into the EAE mice at a dose of 2 ml/kg via the tail vein. Three hours later, the mice were anesthetized with pentobarbital and then perfused with PBS. Spinal cord tissues were collected, and wet weights were measured. Tissues were homogenized in *N*,*N*-dimethylformamide (Sigma). After 24 h of incubation to extract dye at 37 °C, the samples were centrifuged at 21,000×*g* for 30 min. The absorbance of Evans blue in the supernatant was then measured with an Epoch™ Microplate Spectrophotometer (Bio-Tek) at 620 nm and 740 nm. Background absorbance, calculated as -log OD620 = (0.964)(−log OD740) − 0.0357, was subtracted as previously described [23]. Dye concentrations were calculated from a standard curve. The data were expressed as μg/g of tissue weight.

### Quantitative real-time PCR

Total RNA was isolated from lumbar spinal cords using TRIzol reagent (Thermo Fisher Scientific) according to the manufacturer’s instructions. cDNA was synthesized from 300 ng total RNA using ReverTra Ace qPCR RT Master Mix with gDNA Remover (Toyobo, Osaka, Japan). Real-time quantitative PCR was performed using the Applied Biosystems 7500 Real-Time PCR System with SYBR® Green Real-time PCR Master Mix (Toyobo, Osaka, Japan). Actin was measured as a housekeeping gene. The relative gene expression ratio was calculated based on the 2^-deltadeltaCT^ method. Measurements were performed in duplicate. The nucleotide sequences of the primers are listed in Table 1.

**Table 1:** Primers sequences used for RT-PCR

Gene	Primer sequence (5′ to 3′)	
	Forward	Reverse
β-actin	GAGACCTTCAACACCCCAGC	ATGTCACGCACGATTTCCC
Cxcl1	CACAGGGGCGCCTATCGCCAA	CAAGGCAAGCCTCGCGACCAT
Ccl2	CCTGCTGTTCACAGTTGCC	ATTGGGATCATCTTGCTGGT
IL-1β	GCTTCAGGCAGGCAGTATCA	TGCAGTTGTCTAATGGGAACG
VWF	CAGACAGACGCCATCTCCAG	TGCAAGCTGTAGGCAAGCAT

### Measurement of interleukin-1β, IL-17, and IFN-γ protein levels in the spinal cord by enzyme-linked immunosorbent assay

The spinal cords were isolated and homogenized on ice in PBS buffer containing protease inhibitor cocktail (Thermo Fisher Scientific). Lysates were centrifuged at 4 °C for 10 min at 12,000×*g*. Supernatants were collected, and protein concentrations were determined using a BCA protein assay kit (Thermo Fisher Scientific). Protein levels of interleukin-1β (IL-1β), IL-17, and IFN-γ were measured using enzyme-linked immunosorbent assay (ELISA) kits (RayBiotech) according to the manufacturer’s protocols. Samples were read with an Epoch™ Microplate Spectrophotometer (Bio-Tek) at 450 nm. The concentration was determined by comparison to a standard curve.

### Statistical analysis

Values are expressed as the means ± standard error of the mean (SEM). Parametric data were analyzed using Student’s *t* test for two groups and one-way ANOVA followed by Dunnett’s posttest for multiple groups. Nonparametric data were analyzed using the Mann-Whitney *U* test. All statistical analyses were performed using GraphPad Prism 5.0 (GraphPad Software), and *P* < 0.05 was considered significant.

## Results

### Plasma ADAMTS13 activities and VWF levels in EAE mice

**Fig. 1:**
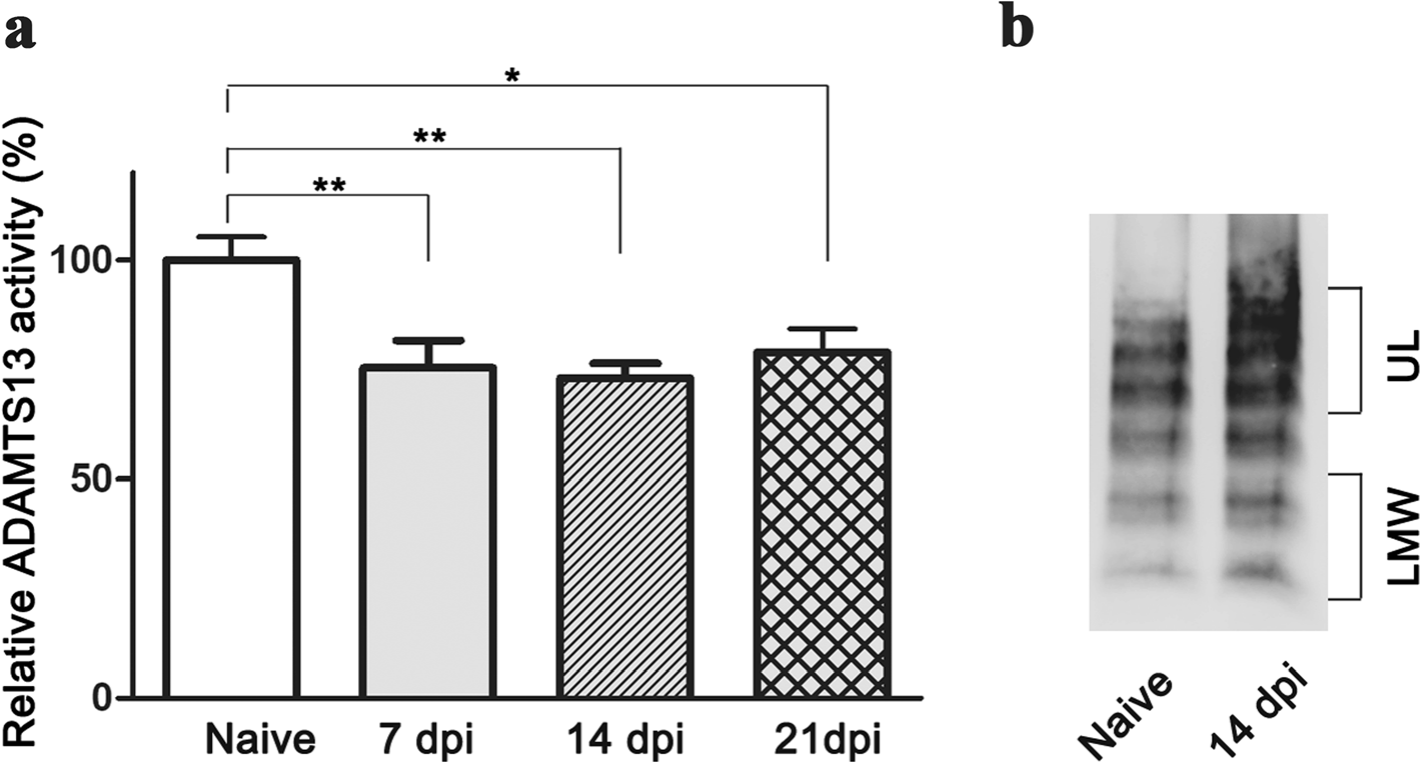
Reduced ADAMTS13 activity and increased VWF levels in the plasma of EAE mice. Plasma was obtained from naïve and EAE mice at different times. **a** Relative ADAMTS13 activities from EAE and naïve mice are shown. **b** VWF levels from the plasma of naïve and EAE mice are shown by western blot. The results are expressed as the mean ± SEM (*n* = 6). Statistical significance was determined by one-way ANOVA followed by Dunnett’s posttest. *P < 0.05; **P < 0.01. *dpi* day post immunization, *LMW* low molecular weight VWF, *UL* ultra large VWF

To evaluate the role of ADAMTS13 in the development of EAE, the kinetics of plasma ADAMTS13 activity were quantified at different time points. Compared to the naïve group, relative ADAMTS13 activity in EAE mice was obviously reduced at 7 dpi (75.4 ± 6.3%; *P* < 0.01), remaining low at 14 dpi (73.2 ± 3.3%; *P* < 0.01). Unpredictably, ADAMTS13 activity at 21 dpi was slightly increased but still lower than that in the naïve group (79.0 ± 5.5%; *P* < 0.05) (Fig. 1a).

Thus far, the only known activity of ADAMTS13 is to cleave large VWF multimers into smaller ones; thus, plasma VWF multimers were assessed in EAE mice. Compared to naïve mice, EAE mice at 14 dpi had a significant increase in the level of ultra large VWF (UL-VWF) (Fig. 1b). Moreover, there was a substantial increase in the level of low-molecular-weight VWF (LMW-VWF) in the plasma of EAE mice at 14 dpi compared to that in naïve mice (Fig. 1b).

### Preventive treatment with ADAMTS13 ameliorates EAE clinical score

**Fig. 2:**
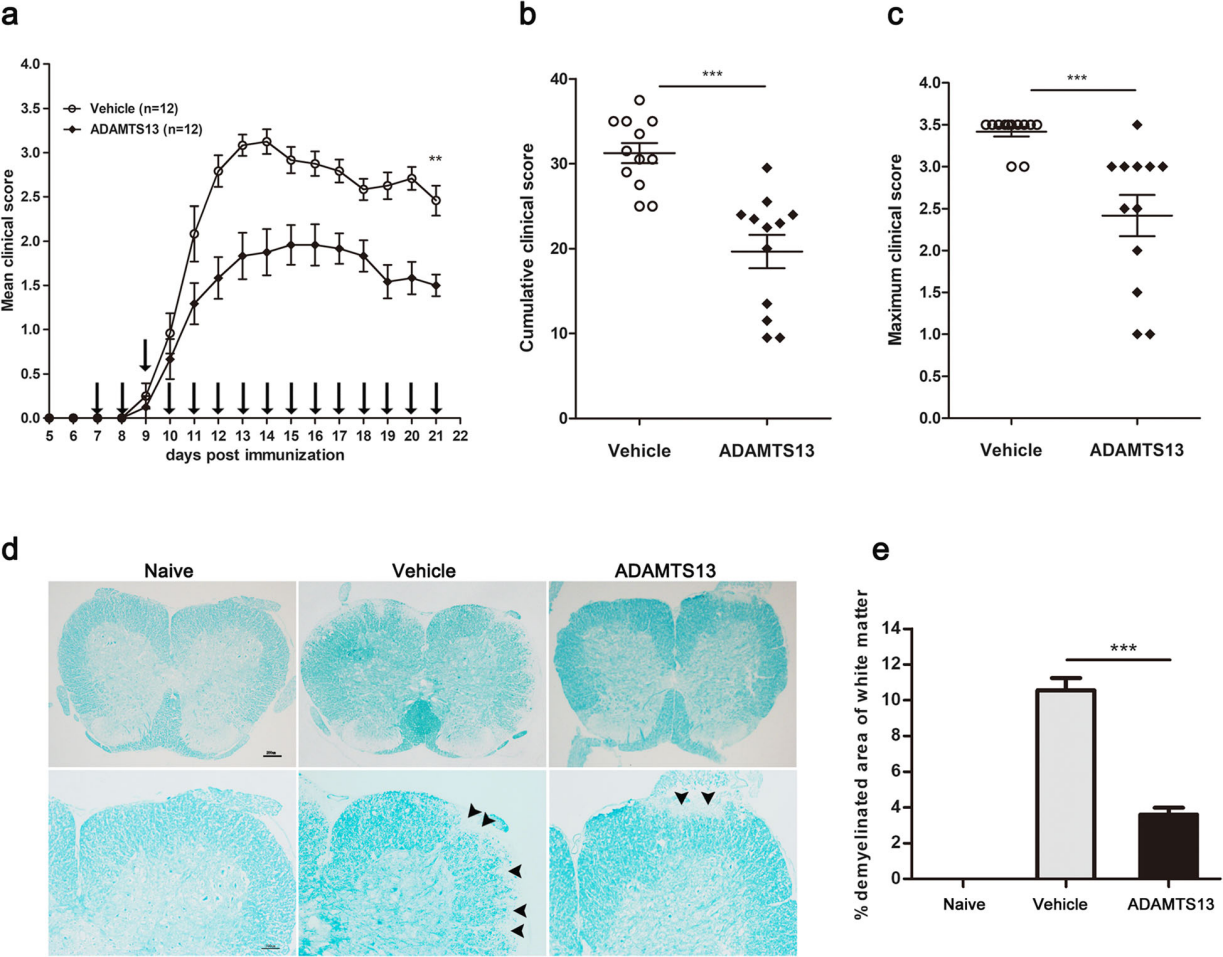
Preventive treatment with ADAMTS13 reduces clinical scores and preserves myelin in EAE mice. ADAMTS13 (50 ng in 2 μl PBS) or vehicle (2 μl PBS) was injected as a preventive treatment into the lateral ventricle of EAE mice every day from 7 dpi to 21 dpi. **a** The daily mean clinical score is shown, and the mean clinical scores at 21 dpi were compared between vehicle and ADAMTS13 group (**P < 0.01). **b** Cumulative clinical scores were summed by adding daily clinical scores from 7 dpi to 21 dpi. **c** The maximum clinical score of every EAE mouse was compared between groups. Data are from a single experiment representative of two independent experiments (**a**–**c**). ADAMTS13- or vehicle-treated EAE mice were sacrificed at 22 dpi, and lumbar spinal cord tissues were harvested. **d** Sections of lumbar spinal cords from EAE mice were subjected to LFB staining for assessment of demyelination and arrowheads show demyelinating lesions. Bars represent 100 μm (the upper panels) and 200 μm (the lower panels). **e** The extent of demyelination was compared between ADAMTS13- and vehicle-treated EAE mice (*n* = 4 each group). Values are expressed as mean ± SEM. Statistical significance was determined by the Mann-Whitney *U* test for clinical scores and by Student’s *t* test for percent demyelination. **P < 0.01; ***P < 0.001

To test our hypothesis that ADAMTS13 may exert protective effects on EAE mice, we injected EAE mice with ADAMTS13 or vehicle from 7 dpi to 21 dpi. The mean clinical score of ADAMTS13-treated mice at 21 dpi was significantly lower than that of vehicle-treated mice (Fig. 2a, Additional file 1). Furthermore, the cumulative clinical score summed from 7 dpi to 21 dpi was significantly reduced by ADAMTS13 treatment (Fig. 2b). There was a reduction in the maximum clinical score (vehicle: 3.4 ± 0.1 vs. ADAMTS13: 2.4 ± 0.2; *P* = 0.0002, Fig. 2c). However, there was no difference in the disease onset day between ADAMTS13-treated mice and the vehicle group. Subsequently, we investigated the preventive effect of ADAMTS13 on demyelination in the spinal cord through LFB staining. The extent of demyelination was much lower in the ADAMTS13-treated mice compared with that in the mice treated with vehicle (Fig. 2d, e).

### Preventive ADAMTS13 treatment reduces the inflammatory burden in the spinal cord of EAE mice

**Fig. 3:**
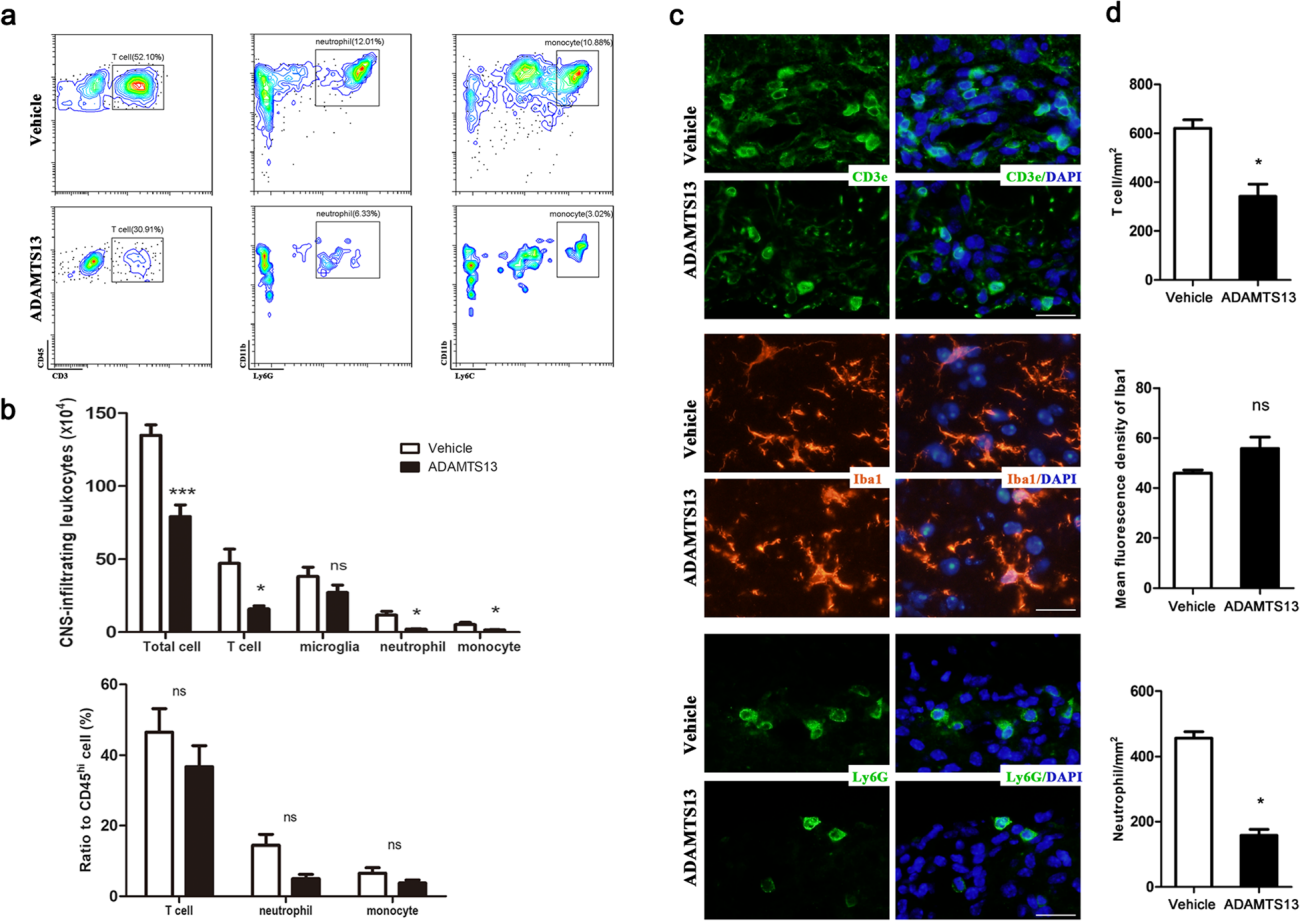
Effect of preventive ADAMTS13 treatment on leukocyte infiltration into the spinal cords of EAE mice. The whole spinal cords from ADAMTS13- or vehicle-treated EAE mice at 22 dpi were isolated and measured by flow cytometry (**a, b**). Leukocyte populations were gated as follows: T lymphocytes (CD45^hi^ CD11b^−^ CD3^+^), microglia (CD45^mild^ CD11b^+^), neutrophils (CD45^hi^ CD11b^+^ Ly6C^int^ Ly6G^hi^), and monocytes (CD45^hi^ CD11b^+^ Ly6C^hi^ Ly6G^−^). **a** Representative plots of leukocyte subsets (pre-gated on CD45^hi^ leukocytes) in the spinal cords from EAE mice treated with vehicle or ADAMTS13. **b** The numbers and frequencies of leukocyte subsets compared to CD45^hi^ leukocytes in the spinal cords of EAE mice (*n* = 6 each group). **c** Representative immunofluorescence images of spinal sections from EAE mice at 22 dpi are shown. Tissue sections were stained with antibodies against CD3 (upper; green, T lymphocytes), Iba1 (middle; red, macrophages/microglia), and Ly6G (bottom; green, neutrophils). DAPI was used to stain the nucleus (blue staining). Bars, 20 μm. **d** Quantitative analysis of immunofluorescence images is shown (*n* = 4 each group). Values are expressed as the mean ± SEM, and Student’s *t* test was used to compare differences between groups. *P < 0.05; ***P < 0.001; *ns* no significance

To determine how ADAMTS13 treatment ameliorated the EAE clinical score, we isolated leukocytes from the spinal cords of EAE mice at 22 dpi and quantified CD45^+^ leukocytes. Both innate and adaptive immune reactions have been believed to take part in MS and EAE pathogenesis [24]. Thus, we analyzed the infiltration of different leukocyte subsets into spinal cords via flow cytometry and the gating strategy is shown in Fig. 3a. The total number of immune cells in the spinal cords of EAE mice was obviously reduced by ADAMTS13 treatment (Fig. 3b). Moreover, the absolute numbers of T lymphocytes, neutrophils, and monocytes decreased clearly in ADAMTS13 group compared to those in vehicle group (Fig. 3b). But the difference of microglia numbers between two groups did not reach statistical significance. Among these CD45^hi^ leukocytes infiltrated into spinal cords, the proportions of T lymphocytes, neutrophils, and monocytes were not significantly reduced by preventive ADAMTS13 administration (Fig. 3b).

We next assessed the localization of infiltrated leukocytes via immunofluorescence staining of frozen spinal cord sections from EAE mice at 22 dpi. Interestingly, cells expressing CD3 (T lymphocyte marker) or Ly6G (neutrophil marker) were consistently detected around blood vessels in the white matter (Fig. 3c). In line with the flow cytometry results, ADAMTS13 administration dramatically reduced the numbers of CNS-infiltrating T lymphocytes and neutrophils, without affecting the microglia marker Iba-1 immunofluorescence intensity (Fig. 3d).

**Fig. 4:**
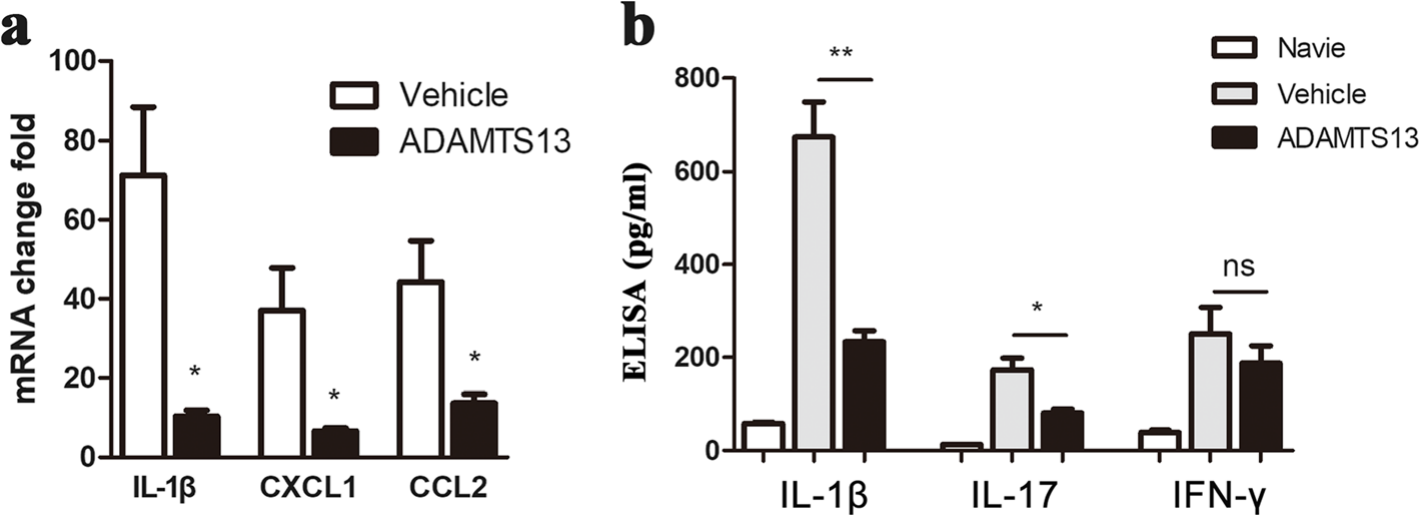
Effect of preventive ADAMTS13 treatment on inflammatory factors in EAE mice. **a** Quantitative RT-PCR analysis of IL-1β, CXCL1, and CCL2 mRNA levels in the spinal cords of ADAMTS13- and vehicle-treated EAE mice at 22 dpi. Data represent fold changes compared to the spinal cords of naïve mice. **b** Supernatants obtained from spinal cord homogenates from naïve, ADAMTS13- and vehicle-treated EAE mice at 22 dpi were subjected to ELISA kits for IL-1β, IL-17, and IFN-γ. The results are expressed as inflammatory factor concentrations, averaged by the total protein contents of the supernatants. Values are expressed as the mean ± SEM (*n* = 5–6) and Student’s *t* test was used to compare differences between groups. *P < 0.05; **P < 0.01; ns, no significance

To further characterize the effect of ADAMTS13 on immune modulation, we conducted an analysis of cytokine and chemokine levels by ELISA and real-time quantitative PCR of the lumbar spinal cord of EAE mice harvested at 22 dpi in the preventive setting. First, compared to vehicle-treated mice, significantly lower levels of CXCL1 and CCL2 expression were detected in ADAMTS13-treated EAE mice, consistent with reduced neutrophil, monocyte, and T lymphocyte infiltration into the spinal cords (Fig. 4a). Second, in mice that received ADAMTS13, IL-1β expression was dramatically reduced at both the RNA and protein levels (Fig. 4a, b). Furthermore, the level of IL-17 protein was dramatically reduced by ADAMTS13 treatment (Fig. 4b). In contrast, IFN-γ levels appeared to be unaffected by ADAMTS13 treatment (Fig. 4b). Collectively, these results suggest a strong anti-inflammatory effect of preventive ADAMTS13 treatment in EAE mice.

### ADAMTS13 decreases VWF levels in spinal cords of EAE mice

**Fig. 5:**
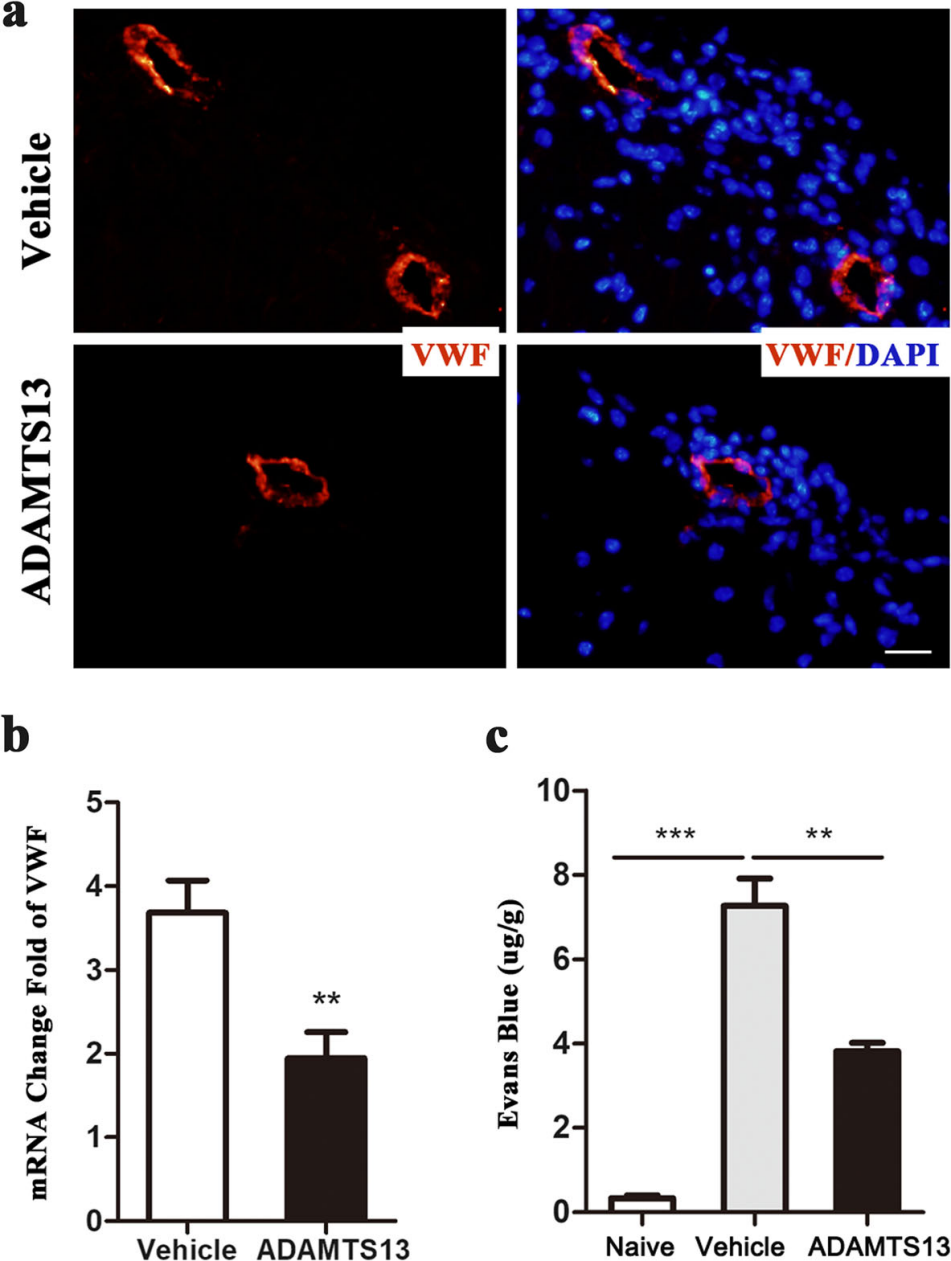
Preventive ADAMTS13 treatment reduced VWF levels in spinal cords and preserved blood-spinal cord barriers (BSCBs) of EAE mice. **a** Tissue sections of spinal sections from EAE mice at 22 dpi were stained with antibodies against VWF (red) and DAPI (blue, nucleus). Bars, 20 μm. **b** Quantitative RT-PCR analysis of VWF mRNA levels in spinal cords of EAE mice at 22 dpi. Data represent fold change compared to the spinal cords of naïve mice. **c** Mice received intravenous an Evans blue injection at 22 dpi, and the amount of Evans blue was averaged by the weights of spinal cord tissue. Values are expressed as the mean ± SEM (*n* = 5–6 each group). Student’s *t* test (**b**) and one-way ANOVA followed by Dunnett’s posttest (**c**) were used to compare differences. **P < 0.01; ***P < 0.001

To date, the only known enzymatic activity of ADAMTS13 is to cleave large VWF multimers into smaller ones. To study the potential mechanisms of ADAMTS13 treatment on cell infiltration into the spinal cords of EAE mice, VWF levels in the spinal cords of EAE mice were assessed. First, immunofluorescence staining showed that VWF-positive areas in the spinal cords of EAE mice were surrounded by numerous infiltrating leukocytes detected via DAPI staining (Fig. 5a). Compared with vehicle, ADAMTS13 preconditioning reduced the VWF levels, along with a reduced number of infiltrating leukocytes (Fig. 5a). Second, increased VWF expression levels were detected by real-time PCR in the spinal cords of EAE mice at 21 dpi compared to levels in the naïve mice (Fig. 5b). VWF expression levels were significantly decreased in EAE mice treated with ADAMTS13 compared with vehicle (Fig. 5b). Together, these results confirmed our hypothesis that ADAMTS13 reduces cellular recruitment into the spinal cords of EAE mice through its proteolytic effect on VWF.

### Preventive ADAMTS13 treatment preserves blood-spinal cord barriers of EAE mice

Recent studies have confirmed the effect of the ADAMTS13-VWF axis on blood–brain barrier (BBB) permeability in mice with stroke and traumatic brain injury [13, 16, 25]. However, whether ADAMTS13 administration preserved the compromised blood-spinal cord barrier (BSCB) in EAE mice is unknown. To assess the degree of BSCB disruption, EAE mice were injected intravenously with Evans blue at 22 dpi, and then, spinal cords were measured for the amount of Evans blue. Statistical tests revealed that Evans blue content in vehicle-treated EAE mice (7.26 ± 0.65 μg/g of tissue) was dramatically higher compared to that in the naïve group (0.34 ± 0.07 μg/g). Furthermore, Evans blue leakage in EAE mice was significantly reduced by ADAMTS13 treatment (3.82 ± 0.20 μg/g of tissue) (Fig. 5c).

### Preventive ADAMTS13 treatment does not affect the peripheral inflammatory response of EAE

**Fig. 6:**
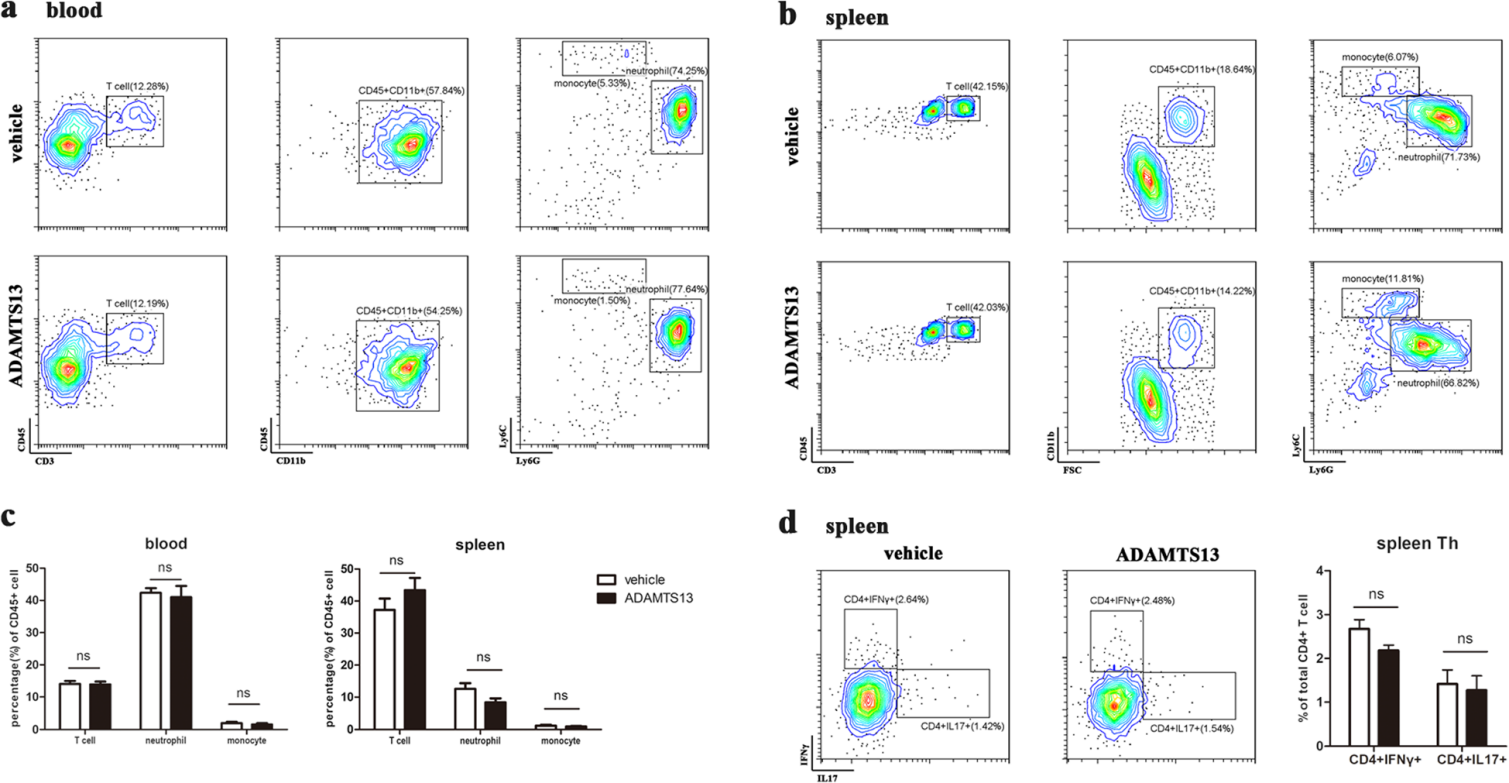
Effect of preventive ADAMTS13 treatment on peripheral inflammation in EAE mice. Cells from the blood (**a**) and spleen (**b**) of ADAMTS13- or vehicle-treated EAE mice at 22 dpi were isolated and measured by flow cytometry. Leukocyte populations were identified as T lymphocytes (CD45^hi^ CD11b^−^ CD3^+^), neutrophils (CD45^hi^ CD11b^+^ Ly6C^int^ Ly6G^hi^), or monocytes (CD45^hi^ CD11b^+^ Ly6C^hi^ Ly6G^−^). **c** Graph illustrating quantitative data for the percentage of inflammatory cells in total CD45^+^ cells in the blood and spleen of mice treated with vehicle or ADAMTS13 at 22 dpi. For intracellular staining, splenocytes were stimulated ex vivo for 5 h and then gated on the CD3^+^CD4^+^ population. **d** The percentages of CD4^+^IFN-γ^+^ and CD4^+^IL-17^+^ cells were assessed. Values are expressed as the mean ± SEM (*n* = 6 each group), and Student’s *t* test was used to compare differences between groups. *P < 0.05; *ns* no significance

Another possible explanation for the reduced CNS inflammatory burden with ADAMTS13 could be the reduced number of peripheral immune cells in the blood and spleen. To test this, we assessed peripheral blood (Fig. 6a) and spleen (Fig. 6b) for T lymphocytes (CD45^hi^ CD11b^−^ CD3^+^), neutrophils (CD45^hi^ CD11b^+^ Ly6C^int^ Ly6G^hi^), and monocytes (CD45^hi^ CD11b^+^ Ly6C^hi^ Ly6G^−^) by flow cytometric analysis. However, ADAMTS13-treated EAE mice had comparable percentages of inflammatory cells to those treated with vehicle (Fig. 6c). Furthermore, we examined the effect of ADAMTS13 on CD4 T cell differentiation via intracellular cytokine staining and flow cytometric analysis of splenocytes at 22 dpi. The data showed a mild decrease in the frequency of IFN-γ-producing Th1 cells in ADAMTS13-treated mice, but the decrease did not reach statistical significance (Fig. 6d). The frequency of IL-17-producing Th17 cells were not different between ADAMTS13-treated and vehicle-treated mice (Fig. 6d). Above all, neither a significant difference in the cell ratio nor a difference in CD4 T cell differentiation toward Th1 and Th17 was detected between ADAMTS13-treated and vehicle-treated EAE mice.

### Protective effects of ADAMTS13 treatment on established EAE mice

**Fig. 7:**
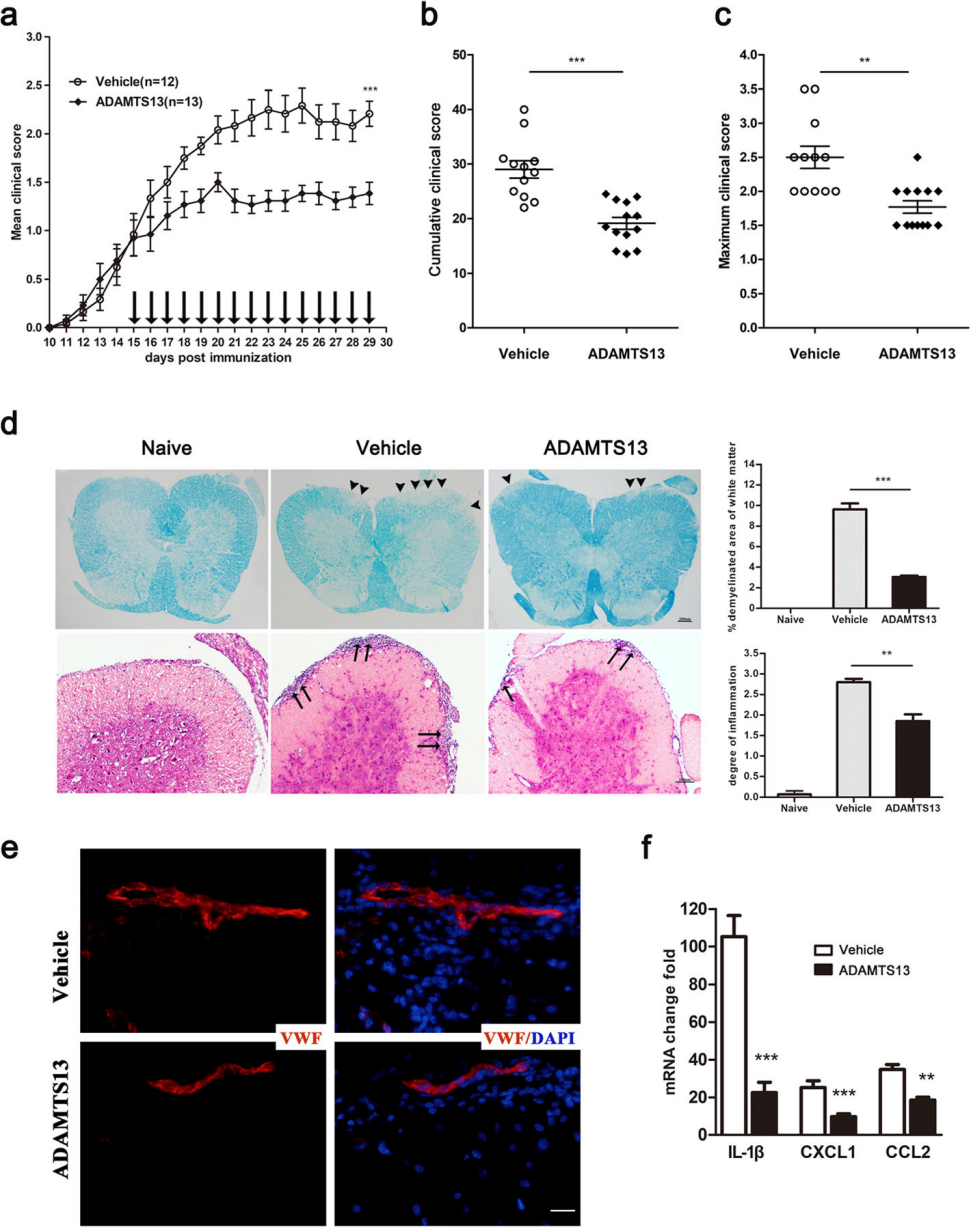
Therapeutic treatment with ADAMTS13 reduces neurological deficits in established EAE mice. Fifty micrograms of ADAMTS13 or vehicle was injected into the lateral ventricle of EAE mice every day from 15 dpi until 29 dpi, and clinical scores were recorded daily. **a** The daily mean clinical score is shown, and the mean clinical scores at 29 dpi were compared between vehicle and ADAMTS13 group (***P < 0.001). **b** Cumulative clinical score was summed by adding daily clinical scores from 15 dpi to 29 dpi. **c** Maximum clinical scores of EAE mice were compared between groups. Statistical significance was determined by the Mann-Whitney U test for clinical scores. **d** Lumbar spinal cord sections of EAE mice at 30 dpi were subjected to LFB (upper panels) and HE (lower panels) staining for assessment of demyelination and inflammation, respectively. The arrowhead shows demyelinating lesions, and the arrow shows the area of inflammation. Scale bars represent 100 μm and 200 μm. The percent of white matter demyelination and the level of inflammatory cell infiltration of the spinal cord were compared between ADAMTS13- and vehicle-treated EAE mice by Student’s *t* test (*n* = 4). **e** Tissue sections of spinal sections from EAE mice at 30 dpi were stained with antibodies against VWF (red) and DAPI (blue, nucleus). Bars, 20 μm. **f** Quantitative RT-PCR analysis of IL-1β, CXCL1, and CCL2 mRNA levels in the spinal cords of ADAMTS13- and vehicle-treated EAE mice at 30 dpi. Data represent fold changes compared to the spinal cords of naïve mice (*n* = 6). Values are expressed as the mean ± SEM. *P < 0.05; **P < 0.01; ***P < 0.001

The finding that plasma ADAMTS13 activity sustained low at 14 dpi suggests the therapeutic potential of ADAMTS13 for EAE. To test this hypothesis, ADAMTS13 or vehicle was injected once the clinical score reached 1 (from 15 dpi to 29 dpi). ADAMTS13 administration markedly reduced the mean clinical score of EAE mice at 29 dpi compared with the vehicle (Fig. 7a). Furthermore, ADAMTS13 treatment also led to a decrease in the cumulative clinical score (from 15 dpi to 29 dpi) (Fig. 7b) and maximum clinical score (vehicle: 2.5 ± 0.2 vs. ADAMTS13: 1.8 ± 0.1; *P* = 0.0011, Fig. 7c). Subsequently, we performed LFB and HE analyses of spinal cord sections from vehicle and ADAMTS13-treated EAE mice at 30 dpi, and representative images of LFB and HE staining are shown in Fig. 7d. Compared to naïve mice (Fig. 7d, left), substantial demyelination of white matter and extensive inflammatory cell infiltration of spinal cord tissue were observed in EAE mice treated with vehicle (Fig. 7d, middle). In contrast, significant reductions in the extent of demyelination and inflammatory cell infiltration were detected in the spinal cords of ADAMTS13-treated versus vehicle-treated mice (Fig. 7d). Moreover, immunofluorescence staining of the spinal cord sections at 30 dpi showed that the VWF level, along with the number of infiltrating leukocytes, was reduced by ADAMTS13 treatment (Fig. 7e). To evaluate the therapeutic effect of ADAMTS13 on immune modulation, we further analyzed cytokine and chemokine levels by real-time quantitative PCR of the lumbar spinal cord of EAE mice harvested at 30 dpi. Compared to vehicle-treated mice, ADAMTS13-treated EAE mice had distinctly lower expression levels of IL-1β, CXCL1, and CCL2 (Fig. 7f), consistent with reduced inflammatory cell infiltration into the spinal cords.

## Discussion

Decreased ADAMTS13 levels were previously identified in the plasma from patients with many conditions, such as systemic inflammation [26, 27], stroke [28], and MS [9]. However, the importance of decreased ADAMTS13 levels in the plasma of MS patients has not been fully assessed, which prompted us to test the role of ADAMTS13 in autoimmune diseases, such as EAE. Here, for the first time, decreased ADAMTS13 activity was detected in the plasma of EAE mice immunized with MOG_35–55_ peptide. Moreover, we demonstrated that exogenous ADAMTS13 treatment attenuated the development and severity of EAE mice. Histopathological analysis showed alleviated demyelination and inflammatory changes in the spinal cords of ADAMTS13 treated EAE mice.

Consistent with decreased ADAMTS13 levels in the plasma from MS patients [9], we demonstrated that MOG_35–55_ immunized EAE mice had decreased ADAMTS13 activity in plasma. However, the mechanism of decreased ADAMTS13 levels and activity in the plasma of MS patients and EAE mice has not been fully assessed. First, Reiter and colleagues reported the inverse correlation between decreased ADAMTS13 activity and increased VWF-related parameters, suggesting a consumption of ADAMTS13 after the desmopressin or endotoxin induced release of higher multimers of VWF [27, 29]. Consistent with this notion, decreased ADAMTS13 activity and upregulated VWF levels in the plasma of EAE mice were observed in our study. Second, several inflammatory cytokines such as IFN-γ, tumor necrosis factor-α (TNF-α), and IL-4, which are markedly elevated in MS patients and EAE mice [22], are known to suppress ADAMTS13 biosynthesis and secretion from hepatic stellate cells and endothelial cells [30]. Additionally, IL-6 was reported to inhibit the VWF-cleaving activity of ADAMTS13 [31]. Finally, a number of recent studies demonstrated that interferon treatment reduced ADAMTS13 levels in MS patients [32, 33]. In contrast, other studies did not discover a connection between interferon treatment in MS and decreased ADAMTS13 levels [9, 34]. In conclusion, it is speculated that the reduced ADAMTS13 activity may result from the consumption of substantial VWF released from activated endothelial cells in MS patients and EAE mice. However, we could not exclude the direct effect of inflammatory cytokines on ADAMTS13 activity. Further studies are required to determine the relationship between interferon treatment and ADAMTS13 levels.

Moreover, we demonstrated that exogenous ADAMTS13 treatment suppressed the development and severity of EAE mice by alleviating demyelination and the inflammatory response in the spinal cord. Our results showed that ADAMTS13 treatment obviously reduced the total number of immune cells in the spinal cords of EAE mice. Although the ratios to CD45^hi^ infiltrating cells were not clearly reduced, the absolute numbers of T lymphocytes, neutrophils, and monocytes were clearly decreased in ADAMTS13 group. In line with the reduced number of immune cells, our study detected significantly lower levels of CXCL1, CCL2, and IL-1β in the spinal cords of ADAMTS13-treated EAE mice. It has been commonly confirmed that not only CD4^+^ T cell but also neutrophils and monocytes play a role in MS and EAE [3, 35, 36]. As CD4^+^ T cell subsets, T helper 17 (Th17) cells and Th1 cells produce IL-17 and IFN-γ, respectively, to mediate inflammation and autoimmunity [3, 37]. In our study, ADAMTS13 treatment evidently reduced the protein levels of IL-17 in the spinal cords of EAE mice, but the decreased levels of IFN-γ were not statistically significant. No change in IFN-γ expression despite significantly reduced T cell in spinal cords might be explained with other IFN-γ producing cells such as natural killer (NK) cells [38]. It has been increasingly recognized that NK cells play a role in EAE [39–41]. Further studies are required to determine whether NK cells in EAE mice are affected by ADAMTS13 treatment.

However, it is not fully understood how ADAMTS13 mitigates inflammatory cells infiltration into the spinal cords of EAE mice. Based on the results of a previous study showing that ADAMTS13 deficiency promotes leukocyte rolling in inflamed venules and extravasation in tissues via increased VWF activity [42], we hypothesize that ADAMTS13 reduces cellular recruitment into the spinal cords of EAE mice through VWF. In our study, increased VWF expression levels were detected in the spinal cords of EAE mice. Moreover, both preventive and therapeutic ADAMTS13 treatment reduced VWF levels, together with decreased leukocytes around VWF-positive areas. In line with our results, numerous studies have reported that ADAMTS13 obviously reduced inflammation in atherosclerosis [20, 43, 44] and stroke [12, 15] via cleaving active VWF. In addition, VWF has been closely associated with platelet adhesion and aggregation, which has recently been reported to play an essential role in EAE pathology by regulating the differentiation and proliferation of CD4 T cells [45, 46]. However, further studies may be required to elucidate whether the protective effect of ADAMTS13 in EAE mice partly depends on the role of activated platelets.

Dysfunction of the blood–brain barrier (BBB) and invasion of activated leukocytes into the CNS are essential for the development of multiple sclerosis and EAE [47]. Moreover, recent studies have confirmed the effect of the ADAMTS13-VWF axis on BBB permeability in models of stroke, traumatic brain injury, and cerebral malaria [13, 16, 25, 48]. For example, studies have reported that intraventricular administration of VWF leads to increased BBB permeability and that ADAMTS13 blocked BBB opening in both ischemic and hemorrhagic stroke mice [13, 16]. Furthermore, compared with WT controls, VWF knockout mice tend to exhibit obviously alleviated BBB permeability in a model of cerebral malaria, hypoxia, and seizures [48, 49]. In our study, the increased BSCB permeability detected in EAE mice was significantly attenuated by ADAMTS13 administration, and this effect might be involved in the action of ADAMTS13 in the mouse model of MS. In contrast, VWF knockout mice were reported to have increased susceptibility to EAE and worse BBB disruption [50]. The possible explanation for these paradoxical results may be that the absence of VWF led to a defect in Weibel-Palade body formation [51], which stores and secretes both pro-inflammatory and anti-inflammatory factors, thus making it difficult to interpret data. The balance between various factors can be disrupted by VWF knockout, resulting in increased BBB permeability. The VWF antibody may be an excellent tool for further investigating VWF function in EAE mice.

Because preventive ADAMTS13 administration before EAE onset is not feasible in the clinical setting, we further confirmed the protective effect of therapeutic administration of ADAMTS13 in EAE mice. However, there are several limitations in our study. First, given that it is technically difficult for us to inject drugs into the tail vein once a day for 2 weeks, ADAMTS13 was administered intracerebroventricularly in our study, which is not feasible in the clinical setting. Second, we found that the percentage of leukocyte subsets in the peripheral immune response and Th1/Th17 cell subsets in the spleen were not affected by ADAMTS13 administration into the lateral ventricle. However, our finding could not completely exclude a potential effect of ADAMTS13 on the peripheral immune response in EAE mice. Third, although decreased ADAMTS13 activity has been characterized, the role of the ADAMTS13-VWF axis in EAE remains to be determined. Therefore, further work including ADAMTS13 knockout will be required to fully elucidate the role of ADAMTS13 in the pathogenesis of EAE. Finally, it remains to be investigated whether processes other than the VWF pathway might be influenced by ADAMTS13.

## Conclusions

ADAMTS13 activity is suppressed in EAE mice. Furthermore, for the first time, this study shows that ADAMTS13 inhibits the CNS immune response, prevents BSCB disruption, and ameliorates demyelination and disease course in EAE mice. Therefore, our study suggests that ADAMTS13 may represent a potential therapeutic strategy for MS patients.

## Supplementary information

**Additional file 1.** Effect of preventive ADAMTS13 treatment on mean clinical score.

## Data Availability

The datasets used and/or analyzed during the current study are available from the corresponding author on reasonable request.

## Abbreviations

BSCB: Blood-spinal cord barriers
CNS: Central nervous system
EAE: Experimental autoimmune encephalomyelitis
ELISA: Enzyme-linked immunosorbent assay
Iba1: Ionized calciumbinding adapter molecule 1
MS: Multiple sclerosis
PBS: Phosphate-buffered saline
VWF: Von Willebrand factor
