# Supplementary figures and images for: ADAMTS13 ameliorates inflammatory responses in experimental autoimmune encephalomyelitis
Source: J Neuroinflammation. 2020 Feb 19;17:67. doi: 10.1186/s12974-020-1713-z (PMC7029584; doi:10.1186/s12974-020-1713-z)

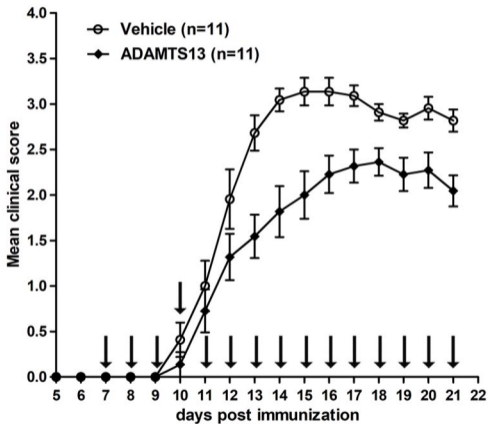

Supplement: Supplementary file 1 — Additional file 1. Effect of preventive ADAMTS13 treatment on mean clinical score. [file 12974_2020_1713_MOESM1_ESM.pdf]
